# Nanomaterial Biosensors in Salivary Diagnosis of Oral Cancer: A Scoping Review

**DOI:** 10.7759/cureus.59779

**Published:** 2024-05-07

**Authors:** Sathya Sethuraman, Karthikeyan Ramalingam, Pratibha Ramani, Kalaiyarasan M

**Affiliations:** 1 Physiology, Saveetha Dental College and Hospitals, Saveetha Institute of Medical and Technical Sciences, Saveetha University, Chennai, IND; 2 Oral Pathology and Microbiology, Saveetha Dental College and Hospitals, Saveetha Institute of Medical and Technical Sciences, Saveetha University, Chennai, IND

**Keywords:** tongue cancer, oral cancer, saliva, nano biochips, nanomaterials, screening tools, diagnostics, oscc biomarkers, biomems, biosensors

## Abstract

Oral cancer is among the highest in the Indian subcontinent. Advanced stages of oral cancer are associated with severe morbidity and higher mortality. Salivary diagnosis is novel and non-invasive. It could be employed on patients even with restricted mouth opening. Hence, an attempt was made to retrieve relevant data regarding this clinically relevant topic.

This article has reviewed metal oxide nanoparticles as a biosensor (BS) in salivary diagnosis for oral cancer. Gold, copper oxide, and carbon nanotubes (CNTs) were used in BS applications. A search from the PUBMED database collection (2004 to 2024) was performed to identify the nanoparticle biomarkers and salivary diagnosis in oral cancer. It revealed 30 articles. All the relevant data was extracted and tabulated in this review.

We have discussed the relevance of these BS in salivary diagnosis with their corresponding clinical parameters and sensitivity. We hope that this review summarizes the available literature on this topic and incites dedicated research in prompt and early diagnosis of oral cancer, which directly influences the quality of life outcomes in such patients.

## Introduction and background

Recent developments in nanotechnology have greatly advanced the creation of innovative methods for the early relief and accurate treatment of many diseases, including oral cancer [1]. Nanomaterials (NMs)-based biosensors (BS) have emerged as a viable platform for developing cancer therapeutic applications, which is the simultaneous diagnosis and treatment of cancer. Technically, in biological microelectromechanical systems (BioMEMS), physiological moieties are controlled for target biomolecule detection, analysis, characterization, and measurement to arrive at a clear conclusion, such as disease or functionality [2].

The recent developments in biosensing through the use of nanotechnology help to overcome the limitations outlined above and have the potential to improve prospective approaches for the detection and diagnosis of oral cancer [3]. Saliva comes into direct contact with oral cancer lesions as a screening tool that is more specific and potentially sensitive than other methods. Additionally, more than 100 salivary biomarkers (DNA, RNA, mRNA, and protein markers) have already been identified, some of the biomarkers include cytokines (IL-8, IL-1b, and TNF-α), defensin-1, P53, Cyfra 21-1, tissue polypeptide-specific antigen, dual specificity phosphatase, spermidine/spermineN1-acetyltransferase, profiling, cofilin-1, transferrin, and many more [4].

Among the most frequently investigated cytokines as candidates for oral squamous cell carcinoma (OSCC) biomarkers, IL-6, IL-8, and TNF-α are present at higher concentrations in the saliva of OSCC patients than in healthy controls. As a result, they may serve as a basis for developing rapid test kits for early recognition of oral cancer [5]. There was a correlation between advanced stages of OSCC and the levels of complement factor H (CFH), fibrinogen alpha chain (FGA), and alpha-1 antitrypsin (SERPINA1) in the patient's saliva [6]. In particular, the combined saliva expression of IL-1-, IL-6, and IL-8, among many other biomarkers evidenced from the literature, represents a valid "trio" based on the sensitivity and specificity of OSCC diagnosis. This is because these three cytokines are known to be involved in the progression of OSCC. Recent research on IL-6 and IL-8 biomarkers in OSCC has revealed that these biomarkers predict OSCC [7].

In this regard, BS have been used for non-invasive early detection. The biological samples are analyzed using BS analytical instruments. They convert biological and chemical responses into electrical signals. BS are classified based on elements that are mostly made up of cells, enzymes, nucleic acids, or antibodies, based on the detectors which can be electronic, pyroelectric, piezoelectric, and pyroelectric, based on the electrical device with a display, processor, and amplification [8]. 

There are numerous methods for saliva diagnosis, including optical techniques like InfraRed, Raman, photoacoustic spectroscopy, surface plasmon resonance BS, capacitive detection, electrochemiluminescence, colorimetry, electrochemical method, and other techniques [9]. Saliva has several advantages over other biological fluids, such as blood and urine, as it is a simple and non-invasive sample. In higher concentrations, saliva also carries the electrochemical communication interaction between the enzyme and electrode to sense the biomolecules. The nanostructure and nanoparticles showed promising results in a high surface volume ratio, as they allow communication between the enzyme and electrodes. The nano BS-based emerging diagnostics and screening tools paved the way for quick, easy, reliable, and robust oral cancer detection tests. Our review article discusses nanoparticles as a BS in salivary diagnosis.

## Review

Systematic reviews should follow PRISMA guidelines with a rationale, specific inclusion and exclusion criteria, database search, methods of extraction, variables and outcome/effect measures, synthesis methods, bias assessment, and certainty of evidence [10]. The inclusion criteria were NM BS utilized for salivary diagnosis in oral cancer or tongue cancer or OSCC. Exclusion criteria were case reports and literature reviews.

A thorough search was conducted in the PubMed database for published manuscripts from 2004 to April 2024. We used the following search terms: ((nanomaterial) AND (Biosensor)) AND ((((saliva diagnosis) AND (Oral cancer)) OR (Tongue Cancer)) OR (oral squamous cell carcinoma)) [11].

**Figure 1:**
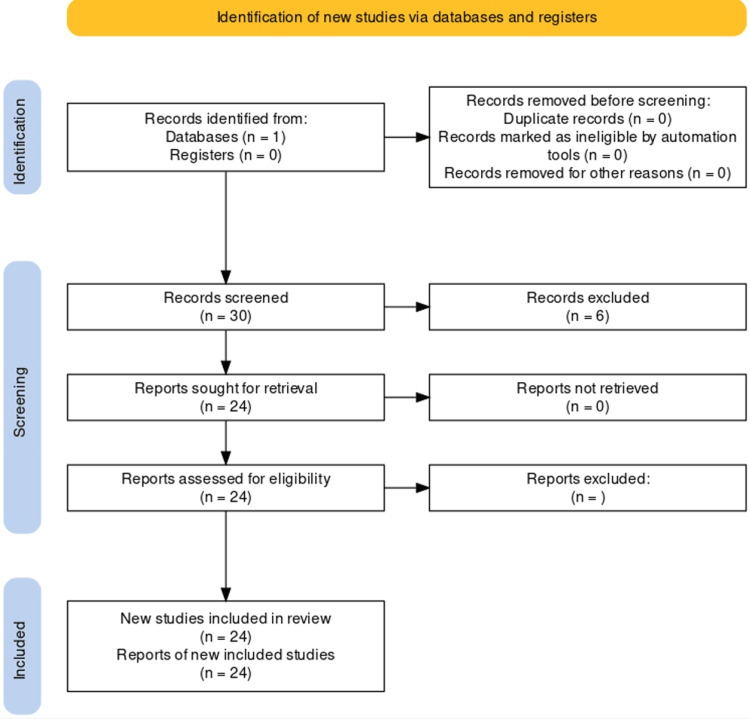
Flow diagram of the article search Flow diagram created with https://estech.shinyapps.io/prisma_flowdiagram/

The PUBMED search revealed only 30 articles. The screening of available articles revealed that a wide array of NMs were utilized in different compositions and structures. Thus, the number of articles retrieved for each nanoparticle BS was extremely limited and a systematic review could not progress further (Figure 1).

Hence, we have attempted a scoping review to identify various NM modalities available for salivary diagnosis in oral cancer. The authors screened the 30 articles and extracted data regarding nanoparticle BS in the salivary diagnosis of oral cancer as mentioned in the articles. 24 articles were included in this review and six were excluded as we could not retrieve the complete article (Figure 1).

All the data was extracted and tabulated with author details, year, NM type, technique, detection range, and advantages. Characterizations of the studies analyzed the NMs along with their role in saliva diagnosis. The description of the techniques and detection range of NM sensor applications are given in Table 1.

**Table 1:** Table summarizing the articles on BS for salivary diagnosis of oral cancer NM, nanomaterials; BS, biosensors; GNRs, gold nanorods; MGPs, magnetic glass particles; QDs, quantum dots; VANTA, vertically aligned carbon nanotube array; Ag, silver; CNT, carbon nanotube; FASA, fully automated saliva analyzer; ALP, alkaline phosphatase; FET, field effect transistor; MBs, magnetic beads; LSPR, localized surface plasmon resonance; SERS, surface-enhanced Raman scattering; CEA, carcinoembryonic antigen; SCCA, squamous cell carcinoma antigen; CIP2A, cancerous inhibitor PP2A; OPC, opal photonic crystal; NIR, near-infrared; anti-EGFR, anti-epidermal growth factor receptor; DL, detection limit

S.No	Author and year	NMs	Techniques	Detection range	Advantages
1	Hu X (2024) [12]	AuNPs@ZIF-8/Cu nano particles	One-step strand displacement reaction	0.1-104 pM	Label-free, simple, and sensitive detection of ORAOV 1 in saliva
2	Li J (2024) [13]	AuNPs@ZIF-8 nanocomposites	Nonenzymatic, electrochemical DNA BS	Wide linear range (1 fM∼1 nM), a low limit of detection (163 aM)	Nondestructive and early screening of oral cancer
3	Li Y (2023) [14]	Hydrophilic PEI ligands are modified on upconversion nanoparticles	OPC-enhanced upconversion fluorescence	Enhanced sensitivity of BS. The limit of detection was 0.1 ng mL^-1^	To detect the CEA in saliva
4	Jiang Y (2023) [15]	β-cyclodextrin/Ti3C2Tx MXenes (β-CD/Ti3C2Tx) as a signal amplifier	Electrochemical sandwich-like immunosensor	0.05 pg mL^-1^ to 20.0 ng mL^-1^	For the detection of SCCA. Applicable in serum samples too
5	Wang X (2022) [16]	AuNPs@HRP@FeMOF	Immune scaffold and FASA	Detection range of 3.1-50.0 ng/mL	Detection of Cyfra21-1, full automation, high integration, time-saving, and low cost
6	Chakraborty D (2022) [17]	GNRs	Nanosized optical transducer technology	0.496-48.4 ng mL^-1^	For sensing oral cancer biomarkers Cyfra 21-1 and CA-125
7	Chaibun (2022) [18]	Sandwich hybridization of the HPV target to the silica-redox dye	Electrochemical DNA BS	1 fM and 1 µM	To detect the high-risk HPV genotypes 16 and 18
8	Tofighi (2021) [19]	Ag nano-ink	Paper-based electrochemical immunosensor	0.0025 to 10 ng/mL	Bioanalysis of Cyfra 21.1 biomarker sensitive, cost-effective, portable, and simple
9	Hafez (2021) [20]	Ag shell around the Au nanobipyramids	ALP multicolor BS	0.1-20 U/L	High selectivity, 98.6% reliability
10.	Jimenez (2019) [21]	MGPs, CdTe/ZnSe core/shell QDs	One-step detection of viral infection, magnetic isolation	1.0x109 (GEq/mL)	Faster and cost-effective for the detection of HPV
11	Qualliotine (2019) [22]	Ultrasound-powered gold nanowire nanomotors	Acoustically powered nanomotors	2.3-times greater intensity	Identifies HPV16 E6 mRNA transcripts extracellularly
12.	Wang (2019) [23]	NIR-II probe named TQTPA	NIR-II imaging, chemo-photothermal therapy	IR-808 under NIR exposure	Good imaging capability, anti-tumor efficacy, biocompatibility, and low systematic toxicity
13	Ding (2018) [24]	VANTA	CIP2A immunosensor	0.24 pg/mL	Detects human CIP2A - an oncoprotein. Rapid cancer screening tests
14.	Kumar (2019) [25]	Yttrium oxide	Impedometric BS	0.33 ng/mL^-1^	Non-invasive detection of CYFRA-21-1 cancer biomarker
15	Zhang (2015) [26]	Silicon nanowires	FET BS	10 fg/mL in PBS and 100 fg/mL in artificial saliva	Multiplexed detection of OSCC
16	Dinish (2014) [27]	SERS nanotag	SERS		Sensitive multiplex detection
17	Wang Z (2013) [28]	Gold electrode	Electrically magnetic-controllable gold electrode junction-probe strategy and MBs	2.2×10 (-19)M	Ease of fabrication, operational convenience, short analysis time, good stability, and re-usability
18	Gong T (2013) [29]	Gold nanoparticles	Darkfield microscopy technique plasmon scattering probes	100 nm spectral separation in absorption bands	Seven types of plasmon scattering probes
19	Austin (2011) [30]	Ag plasmonic nanoparticles	Plasmonic scattering property and nuclear-targeting property		Real-time behavior of human oral squamous carcinoma, induction of apoptosis in HSC-3 cells
20	Zhou W (2011) [31]	Ag nanoparticle array	LSPR nanosensor	Serological diagnosis or molecular diagnosis of SCC	Sense serum p53 protein in head and neck SCC
21	Malhotra (2010) [32]	CNT	Ultrasensitive electrochemical immunosensor for human IL-6	The DL of 0.5 pg19.3 nA mL mL(-1)	SWNT immunosensors combined with multilabel detection have excellent promise for detecting IL-6 in research and clinical applications
22	Bajaj (2009) [33]	Nanoparticle-polymer sensor arrays	Chemical nose/tongue approach		Differentiates normal, cancerous, and metastatic cells
23	Kah (2007) [34]	Gold nanoparticles	Self-assembled SERS		Easy and effective differentiation between normal and oral cancer patients
24	Huang (2006) [35]	Gold nanorods conjugated to anti-EGFR)	Laser photothermal therapy NIR laser pulse. Molecular imaging and photothermal cancer therapy	NIR region (650-900 nm)	Effective for early diagnostics as well as treatment

Hu et al. presented an electrochemical BS based on Cu2+-doped zeolitic imidazolate frameworks and gold nanoparticles. It is a single-step sensitive test to detect the ORAOV 1 providing more reliable results [12]. For the early detection of non-destructive oral cancer, ultra-sensitive, non-enzymatic AuNPs@ZIF-8 nanocomposite was developed [13]. Li et al. proposed an opal photonic crystal (OPC)-enhanced upconversion fluorescence to detect the carcinoembryonic antigen (CEA) in saliva by applying fluorescence. The upconversion nanoparticle enhances the sensitivity of BS. It is a valuable tool in early detection and monitoring and can be done by the patient at their residence [14]. 

Wang et al. fabricated a single-step FASA by non-invasive detection of Cyfra 21-1 in saliva. It offers an efficient and potential instrument for point-of-care cancer screening [16]. Chakraborty et al. highlight the gold nanoparticles use employing the nanosized optical transducer technology for sensing oral cancer biomarkers Cyfra 21-1 and CA-125. The detection range was 0.496-48.4 ng mL [17]. To detect the high-risk HPV genotypes 16 and 18, Chaibun T et al. have used the sandwich hybridization of the HPV target to the silica-redox dye. The electrochemical DNA BS has a detection range of 1 fM and 1 µM [18]. Tofighi et al. used a cost-effective, portable, and simple paper-based electrochemical immunosensor using Ag nano-ink for bioanalysis of Cyfra 21.1 and the linear range was 0.0025 to 10 ng/mL [19]. Hafez et al. found a versatile alkaline phosphatase (ALP) multicolor BS that detects ALP in the saliva. It is a sensitive and reliable method with a reproducible rate of 98.6% [20]. Jimenez AM et al. proposed one-step detection of viral infection by magnetic isolation. It was faster and more cost-effective in the detection of HPV employing magnetic glass particles (MGPs) and CdTe/ZnSe core/shell quantum dots (QDs) [21]. Jiang et al. constructed a simple but effective electrochemical sandwich-like immunosensor with β-cyclodextrin/Ti3C2Tx MXenes (β-CD/Ti3C2Tx) as a signal amplifier. It is effective in detecting the squamous cell carcinoma antigen (SCCA). It was applicable in serum samples too [15]. Invitro studies were conducted by Qualliotine et al. with ultrasound-powered gold nanowire nanomotors to identify HPV16 E6 mRNA transcripts extracellularly [22]. Ding et al. proposed a vertically aligned carbon nanotube array (VANTA) CIP2A. Immunosensor detects human cancerous inhibitor PP2A (CIP2A) - an oncoprotein. It is a rapid cancer screening test [24].

Kumar et al. used Yttria-modified nanostructures and Zhang et al. used silicon nanowires for oral cancer diagnosis [25,26]. Dinish et al. developed a multiplex detection of cancer biomarkers by combining the surface-enhanced Raman scattering (SERS) technique with a hollow-core photonic crystal fiber (HCPCF) [27]. Wang et al. suggest an electrically magnetic-controllable gold electrode junction-probe strategy, and magnetic beads (MBs) is a non-invasive technique that possesses ease of fabrication, operational convenience, short analysis time, good stability, and re-usability [28]. Gong et al. used label-free gold probes for dark field imaging of cancer cells [29]. Austin et al. reported that plasmonic silver nanoparticles had a role in programmed cell death, plasmonic scattering property, and nuclear-targeting properties [30]. The first clinical application of LSPR (localized surface plasmon resonance) nanosensor was postulated by Zhou et al. in 2011 and was used to sense serum p53 protein in serological or molecular diagnosis of SCC [31]. Malhotra et al. have investigated the ultrasensitive electrochemical immunosensor for human IL-6 employing the carbon nanotube (CNT). These immunosensors correlated remarkably with traditional enzyme-linked immunosorbent assays (ELISA), demonstrating the accuracy with which they assessed secreted IL-6 in HNSCC cells [32].

Bajaj et al. investigated the nanoparticle-polymer sensor arrays based on a chemical/nose approach to differentiate between normal, cancerous, and metastatic cells [33]. Kah suggested gold nanoparticles can undergo surface plasma resonance. The Raman scattering effect is employed with the activated gold nanoparticles. SERS spectra of saliva could be distinguished from normal and oral cancer samples. SERS-based saliva assay could be useful for the earlier detection of oral cancer [34]. Huang et al. postulated photothermal destruction of cancer cells using gold nanoparticles conjugated to anti-epidermal growth factor receptor (anti-EGFR) for the early detection of oral cancer [35]. Poonia et al. summarized nanotechnology in oral cancer in their detailed review [36]. Ge et al. used chip-based microsensors for the identification of micro-RNAs [37]. Song et al. suggested that the three-dimensional carbon nanotubes (3DN-CNTs) sensor had a high chance of precisely diagnosing OSCC using the Cyfra 21-1 biomarker [38].

Singhal et al. have discussed NMs [2]. They have tremendous diagnostic potential because of their high surface-to-volume ratio and quantum confinement phenomenon and enhance the detection limit of clinically relevant biomolecules in biofluids. It can be developed from a bench-on platform to a point-of-care (POC) screening device. The NM-based BS fabrication technology has also simplified and improved the diagnosis of oral cancer. Chakraborty et al. have suggested that nanosized optical transducer technology can be further converted into miniaturized biochips and can be deployed in clinics as a nanoparticle-based point-of-care diagnostic adjunct [17].

Despite the inspiring findings of many of the included articles, only a few NMs salivary biomarkers are used as valid techniques for oral cancer. We have attempted to summarize the most recent research on BS for NMs detection. Dedicated clinical trials are needed to find the best approach for the accuracy, affordability, and usability of such nanoparticles in effective oral cancer diagnosis.

## Conclusions

We have reviewed the NMs used in salivary diagnosis for oral cancer using biosensor analytical methods. We have found out that limited articles are present in this field and various nanoformulations are being experimented with. Non-invasive analysis of salivary samples with the highly sensitive and specific determination of oral cancer is the key to improving the quality of life in those patients. Early and accurate diagnosis is vital for enhancing patient outcomes. The development of salivary-based point-of-care devices will enhance disease identification and initiate prompt referral.
